# Carbapenemase-producing *Acinetobacter* spp. in Cattle, France

**DOI:** 10.3201/eid1803.111330

**Published:** 2012-03

**Authors:** Laurent Poirel, Béatrice Berçot, Yves Millemann, Rémy A. Bonnin, Glenn Pannaux, Patrice Nordmann

**Affiliations:** Hôpital de Bicêtre, Le Kremlin-Bicêtre, France (L. Poirel, B. Berçot, R.A. Bonnin, P. Nordmann);; Hôpital Lariboisière, Paris, France (B. Berçot);; Université Paris-Est, Maisons-Alfort, France (Y. Millemann, G. Pannaux)

**Keywords:** carbapenemase, OXA-23, cattle, Acinetobacter, carbapenem-hydrolyzing class D β-lactamases, France, bacteria

**To the Editor:** Multidrug resistance in bacteria isolated from animals is an emerging phenomenon, mirroring what is happening among humans. During the past decade, expanded-spectrum β-lactamases in *Enterobacteriaceae* from humans (*1*) and animals (*2*) worldwide have been reported. Among humans, as a consequence of this high rate, use of carbepenems is increasing selection pressure; carbapenem-resistant gram-negative organisms are increasingly reported, including carbapenemase-producing *Enterobacteriaceae* and *Acinetobacter* spp (*3*).

The most commonly acquired carbapenemases identified in *Acinetobacter* spp. correspond to carbapenem-hydrolyzing class D β-lactamases (*3*). In particular, the worldwide spread of OXA-23–producing *A. baumannii* is considered a serious threat; those strains are frequently involved in nosocomial outbreaks for which therapeutic options are extremely limited (*3**,**4*). Our study objective was to evaluate the possible occurrence of carbapenemase-producing gram-negative bacteria in dairy cattle in France.

In August 2010, at a dairy farm 30 km from Paris, France, rectal swabs were collected from 50 cows. Samples were precultured in buffered peptone water and incubated for 18 h at 37°C. Cultures were inoculated by streaking 100 μL of the suspensions onto Drigalski agar plates (bioMérieux, Balmes-les-Grottes, France) containing 1 μg/mL of imipenem to select for carbapenem-resistant gram-negative isolates. Of the 50 samples, 9 produced growth on imipenem-containing plates. All colonies tested (10 colonies/sample) by using the API 20 NE (bioMérieux) system were first identified as *A. lwoffii*. Molecular techniques based on sequencing of the *gyrA*, *gyrB*, and *rpoB* genes (*5*) enabled more precise identification and indicated that all isolates belonged to the *Acinetobacter* genomospecies (DNA group) 15TU, which is known to be phylogenetically related to *A. lwoffii* and which has been reportedly isolated from sewage, freshwater aquaculture habitats, trout intestines, and frozen shrimp (*6*).

One colony per sample was retained for further investigation (isolates BY1 to BY9). Susceptibility testing and MIC determinations were performed by disk-diffusion assay (Sanofi-Diagnostic Pasteur, Marnes-la-Coquette, France) and Etest (AB bioMérieux, Solna, Sweden) (Table). All isolates except 1 were resistant to penicillins, combinations of penicillins and β-lactamase inhibitors, and carbapenems but susceptible to cefotaxime and of reduced susceptibility to ceftazidime. Isolate BY1 showed higher MICs for carbapenems (Table). In addition, all isolates were resistant to tetracycline, kanamycin, and fosfomycin and remained susceptible to fluoroquinolones, chloramphenicol, gentamicin, amikacin, tobramycin, and sulfonamides. Susceptibility profiles of 3 *Acinetobacter* genomospecies 15TU reference strains showed that they were fully susceptible to penicillins, carbapenems, tetracycline, and kanamycin.

**Table:** Antimicrobial drug MICs for *Acinetobacter* genomospecies 15TU isolates from cows and reference strains, France, August 2010

Drug class	MIC, μg/mL				
	*Acinetobacter* genomospecies 15TU			Reference strain	
	BY1	BY2–BY9		NIPH 2171	NIPH 899
Penicillins and combinations					
Amoxicillin	>256	128–256		4	4
Amoxicillin + CLA	>256	128–256		4	4
Cephalosporins					
Cefoxitin	32	16–32		16	16
Cefotaxime	32	16–32		8	6
Ceftazidime	32	16–32		16	16
Cefepime	16	4–16		4	4
Monobactam (aztreonam)	64	32		32	16
Carbapenems					
Meropenem	16	2–4		0.5	0.5
Imipenem	>32	4–6		0.25	0.25
Doripenem	8	2–4		0.5	0.5
Cyclines					
Tetracycline	>256	>256		0.5	0.5
Tigecycline	0.064	0.047–0.064		0.047	0.125
Quinolones (ciprofloxacin)	0.5	0.5		0.25	0.25
Aminoglycosides					
Gentamicin	0.5	0.25–0.5		0.25	0.25
Kanamycin	>256	>256		0.5	0.5
Sulfonamides	4	4		4	>256

*CLA, clavulanic acid (4 μg/mL).

Clonal diversity between the isolates was assessed by pulsed-field gel electrophoresis (*5*), which showed 6 distinct genotypes. Isolate BY1 corresponded to a single clone (data not shown), which indicated that the occurrence of *Acinetobacter* genomospecies 15TU strains among these animals was not the result of dissemination of a single clone.

PCR detection and sequencing of genes that encode carbapenem-hydrolyzing class D β-lactamases (*5*) showed that the 9 *Acinetobacter* genomospecies 15TU isolates harbored a *bla*_OXA-23_ gene, whereas the 3 reference strains remained negative. Sequencing confirmed that all isolates expressed β-lactamase OXA-23, which is known to be widespread in *A. baumannii*.

Mating-out assays and plasmid electroporation assays were performed by using *bla*_OXA-23_–positive *Acinetobacter* spp. isolates as donors and rifampin-resistant *A. baumannii* BM4547 isolates as a recipient strain (*5*); however, these assays were unsuccessful. Plasmid DNA analysis (*5*) gave uninterpretable results, with DNA degradations.

The genetic structures surrounding the *bla*_OXA-23_ gene were investigated by PCR mapping (*7*), which identified transposon Tn*2008* in isolate BY2 only. Tn*2008* is a major vehicle for the spread of the *bla*_OXA-23_ gene in *A. baumannii* in the People’s Republic of China (*8*) and the United States (*9*). In the other isolates, the IS*Aba1* element of Tn*2008* had been truncated by a novel insertion sequence termed IS*Acsp2* (www-is.biotoul.fr).

The dairy farmer indicated that most animals from which OXA-23 producers had been identified had received antimicrobial drugs in the previous weeks. Although 1 animal had received amoxicillin-clavulanate, most of the others had been given oxytetracycline and neomycin to treat mastitis.

β-lactamase OXA-23 is a common source of carbapenem resistance in *A. baumannii (**5**)*. Infections with multidrug-resistant OXA-23–producing *A. baumannii* or *A. junii* have been reported from hospitals but not from the community. Our study showed that OXA-23–producers in particular, and carbapenemase producers in general, may be isolated from animals. Among the hypotheses that could explain the selection of this carbapenemase, use of penicillins or penicillin–β-lactamase inhibitor combinations could create selective pressure for β-lactamases because OXA-23 does confer, in addition to decreased susceptibility to carbapenems, a high level of resistance to those compounds. We have previously shown that *A. radioresistens*, an environmental species, was the progenitor of the *bla*_OXA-23_ gene (*10*). Studies are needed to determine to what extent and at which locations *Acinetobacter* genomospecies 15TU and *A. radioresistens* might co-reside and therefore where the *bla*_OXA-23_ gene exchange might have occurred.
